# Accounting for Dynamic Fluctuations across Time when Examining fMRI Test-Retest Reliability: Analysis of a Reward Paradigm in the EMBARC Study

**DOI:** 10.1371/journal.pone.0126326

**Published:** 2015-05-11

**Authors:** Henry W. Chase, Jay C. Fournier, Tsafrir Greenberg, Jorge R. Almeida, Richelle Stiffler, Carlos R. Zevallos, Haris Aslam, Crystal Cooper, Thilo Deckersbach, Sarah Weyandt, Phillip Adams, Marisa Toups, Tom Carmody, Maria A. Oquendo, Scott Peltier, Maurizio Fava, Patrick J. McGrath, Myrna Weissman, Ramin Parsey, Melvin G. McInnis, Benji Kurian, Madhukar H. Trivedi, Mary L. Phillips

**Affiliations:** 1 Department of Psychiatry, University of Pittsburgh School of Medicine, Pittsburgh, Pennsylvania, United States of America; 2 UT Southwestern Medical Center, Department of Psychiatry, Dallas, Texas, United States of America; 3 Department of Psychiatry and Behavioral Science, Stony Brook University, Stony Brook, New York, United States of America; 4 Department of Psychiatry, Massachusetts General Hospital, Boston, Massachusetts, United States of America; 5 NY State Psychiatric Institute, Therapeutics Depression Evaluation Service, New York, New York, United States of America; 6 Department of Psychiatry, Columbia University College of Physicians and Surgeons, New York, New York, United States of America; 7 Functional MRI Laboratory, University of Michigan, Ann Arbor, Michigan, United States of America; 8 Department of Radiology, Stony Brook University, Stony Brook, New York, United States of America; 9 Department of Psychiatry, University of Michigan School of Medicine, Ann Arbor, Michigan, United States of America; University Zurich, SWITZERLAND

## Abstract

Longitudinal investigation of the neural correlates of reward processing in depression may represent an important step in defining effective biomarkers for antidepressant treatment outcome prediction, but the reliability of reward-related activation is not well understood. Thirty-seven healthy control participants were scanned using fMRI while performing a reward-related guessing task on two occasions, approximately one week apart. Two main contrasts were examined: right ventral striatum (VS) activation fMRI BOLD signal related to signed prediction errors (PE) and reward expectancy (RE). We also examined bilateral visual cortex activation coupled to outcome anticipation. Significant VS PE-related activity was observed at the first testing session, but at the second testing session, VS PE-related activation was significantly reduced. Conversely, significant VS RE-related activity was observed at time 2 but not time 1. Increases in VS RE-related activity from time 1 to time 2 were significantly associated with decreases in VS PE-related activity from time 1 to time 2 across participants. Intraclass correlations (ICCs) in VS were very low. By contrast, visual cortex activation had much larger ICCs, particularly in individuals with high quality data. Dynamic changes in brain activation are widely predicted, and failure to account for these changes could lead to inaccurate evaluations of the reliability of functional MRI signals. Conventional measures of reliability cannot distinguish between changes specified by algorithmic models of neural function and noisy signal. Here, we provide evidence for the former possibility: reward-related VS activations follow the pattern predicted by temporal difference models of reward learning but have low ICCs.

## Introduction

It is essential to improve the test-retest reliability of the blood oxygenation level dependent (BOLD) signal both to provide a deeper understanding of individual differences in context-dependent neural responses, as well as a meaningful interpretation of functional neural circuitry changes across time. Currently, obtaining reliable neural activation appears somewhat elusive, with some studies reporting strikingly consistent activations across time between participants (e.g. [1]) and other studies reporting low test-retest reliability (e.g. [2]). As might therefore be expected, the literature as a whole seems to align somewhere between the two, with a modest (‘fair’) reliability: the pooling of studies reporting Intra-Class Correlations (ICCs), a conventional metric of data reliability, yielded an average ICC of around 0.5 [3]. Notably, such levels of reliability would be unacceptable in many other fields of scientific investigation, and this has tempered optimism about the use of functional magnetic resonance imaging (fMRI) to provide meaningful insight into individual differences [4].

Several approaches have been utilized to improve reliability of fMRI methods including a careful attention to the acquisition parameters within and across scanners, especially in multisite studies. Beyond image acquisition, another limiting factor of reliability may be the statistical analysis pipeline employed [5, 6]. Mis-specification in the modeling of the BOLD response, perhaps due to systematic differences in the haemodynamic response function (HRF: [7]) or the problematic consequences of physiological noise [8] may be serious impediments to the reliability and reproducibility of patterns of neural activation. We recently demonstrated that improvement in the test-retest reliability of response in some brain regions can be achieved by correcting for both HRF and BOLD response modeling [9]. However, emotion-related amygdala activation was generally unreliable, as assessed by a conventional measure of reliability (ICCs: (see also [2, 10, 11]), and was not consistently improved by alternative modeling strategies. This was observed despite the capability of the procedure to reveal clinically relevant individual differences in amygdala activation [12]. Among several possible explanations for this discrepancy, one likely explanation is that as predicted by previous empirical [13, 14] and theoretical conceptions of amygdala function [15, 16], amygdala activation may change dynamically during the experimental paradigm itself.

A good place to investigate further the possibility of dynamic change in neural response in specific regions of interest is to examine reward-related activation in the ventral striatum (VS). Considerable empirical evidence has accumulated on activation of this region during reward processing and hypotheses about the VS have been sharpened by numerous studies. A dominant hypothesis of reward-related activation in the VS is that it follows predictions according to the temporal difference (TD) model of learning [17]. This model posits that reward-related activation occurs when there is a deviation from expectation, and that it reflects whether an event is better or worse than expected (a ‘signed prediction error’). Importantly, with training, the same signal becomes coupled to the earliest reliable predictor of reward, e.g., a cue predicting future reward [18, 19]. Concurrently, the signal coupled to the outcome itself—the predicted reward—diminishes, unless the reward is surprisingly increased. From the perspective of reliability, however, activation that fluctuates as a result of the process of conditioning would be expected to be difficult to reproduce, and thus the ventral striatal response to prediction error is likely to be non-stationary across time. Thus, although variation in activation levels from the first to second scanning session will lead to low estimates of reliability using ICCs or similar measures, such variation may be *predictable* insofar as it follows what we would have expected from a learning mechanism.

In a previous fMRI study of a reward paradigm (based on [20, 21]), we examined the neural response in VS to outcome-coupled positive prediction errors in healthy control (HC) participants—events when an outcome is more rewarding (i.e. has a greater monetary value) than was expected [22]. We also observed a negative correlation between the magnitude of this response and the activation in the VS associated with a prior cue which signaled the likelihood of obtaining reward. This negative relationship follows from the predictions of the TD model: the process of conditioning should reduce outcome-coupled prediction error- (PE) related and increase cue-coupled reward expectancy- (RE) related activation (see also [23]). Individuals with relatively high learning rates would then show relatively increased RE- and reduced PE-related VS activation. We would also expect this pattern to generalize to a second testing session, i.e., RE activations would continue to increase and PE activations would fall further. Although learning is not an explicit requirement of the guessing task used below, the pattern of our previous neural findings implies this type of process, and we expected reward-related brain regions to track the ongoing reward contingencies as the task progresses. Ongoing computation within these regions would lead to a representation of the expected value of task events, and may concord with a ‘critic’ [24], which encodes changes in the future expected value (prediction errors) independent of the action policy selected. Moreover, given the behavioral policies on a guessing task are unconstrained, a signal reflecting a critic may be more easily identified across participants than one that reflects action policy.

Abnormal VS activation during reward processing has been repeatedly reported in major depressive disorder (MDD). However, variability of findings across cohorts and paradigms, and the presence of robust activation in response to some reward-related stimuli (e.g. [22, 25, 26]) might suggest an alteration of the normal pattern of prediction error-related modulation of VS activation [27–29], rather than a generalized hypo-activation of the region. Further examination of the extent to which PE-related modulation of VS activation is disrupted in depressed individuals with MDD, and the extent to which this may be ameliorated by antidepressant treatment response [30], is an important area of study in mood disorders research. Furthermore, longitudinal study of VS activation during reward processing in MDD individuals may represent a useful paradigm for identifying biomarkers predicting antidepressant treatment response in MDD. Prior to such study, however, it is necessary to establish the properties of normal longitudinal VS response during reward processing. If the VS activations to reward-related stimuli do indeed change dynamically with repeated testing, analysis of such data for the purpose of defining biomarkers would thus need to focus on the effect of pathology or treatment on the predicted extent of change, rather than on deviations from an expected point estimate.

The present report addresses three aspects of reliability: 1) Evaluation of test-retest reliability of VS activation, 2) Evaluation of the consistency of reward-related activation across four data acquisition sites and 3) Evaluation of the relationship between test-retest reliability and data quality. With regard to the first aim, VS response during PE and RE processing at two time points (1 week apart) was examined in HC using a reward paradigm employed in previous studies. We also examined the test-retest reliability of the anticipation-related activation in the occipital cortex, given our previous findings that occipital responses during an emotion processing task were relatively stable and reliable over time [9]. This occipital cortex activation thus provided a useful control measure. Analyses of the whole brain activation maps were also conducted.

With regard to the second aim, the impact of variation in data quality in and across the four data collection sites on fMRI test-retest reliability was examined. The analysis strategy was derived from a previous study that used the same task, and a comparison of the two studies was performed [22]. With regard to the third aim, we included data that would normally have been excluded due to excess motion, low signal to noise ratio (SNR), artifacts or poor coverage, to enhance the variability of data quality within the sample. We derived a metric of data quality using factor analysis. The reasoning was threefold: first, comparing the reliability of relatively low and high quality data should provide information about how far test-retest reliability is affected by variation in data quality. We were also able to test whether a metric of data quality could be used to weight data points based on their precision and improve regression model estimates. Finally, of most relevance to the EMBARC study, an assessment of the effect of data quality on fMRI reliability has implications for minimum standards of data acquisition for the use of biomarkers in longitudinal, treatment prediction studies.

## Methods

### Participants

HC participants were recruited as part of the Establishing Moderators and Biosignatures of Antidepressant Response in Clinical Care (EMBARC) study, a multi-site longitudinal study aiming to identify neuroimaging and other moderators of treatment response in depressed individuals with MDD (Trivedi et al., in press; [31]). Forty healthy individuals were tested twice, one week apart, in one of four sites (10 participants per site). A group of MDD participants were also recruited and tested at the same sites (n = 148; 97 females; mean age = 37.11; SD = 12.93), but data from this cohort are otherwise not described in the present report. Of the 40 healthy individuals, three participants were excluded—one due to a missing time 1 scan, one due to severe ghosting, and one due to a very low SNR (41) on one scan. This left a final sample of 37 (mean age: 38.03, SD: 15.27; 22 (59%) female). Written informed consent was obtained for all participants in accordance with the Declaration of Helsinki. Enrollment of participants was approved by the UT Southwestern Institutional Review Board, New York State Psychiatric Institute Institutional Review Board (Columbia University), Partners Human Research Committee (Massachusetts General Hospital and McLean Hospital), University of Michigan Medical School Institutional Review Board, and Stony Brook University Institutional Review Board. Additionally, approval to analyze data was obtained from the University of Pittsburgh Institutional Review Board.

Participants were recruited for the study if they were between the ages of 18 and 65. Exclusion criteria included a Quick Inventory of Depressive Symptomatology—Self Report (QIDS-SR) score of 8 or above, lifetime history for MDD, psychotic depression bipolar (I, II, or NOS) disorder, schizoaffective disorder, schizophrenia, other Axis I psychotic disorders, and/or any current Axis I or II diagnoses; presence of DSM-IV criteria for substance dependence in the last 6 months, except for nicotine, or substance abuse in the last 2 months, and/or the presence of a positive urine drug screen at test; currently actively suicidal or considered a high suicide risk; presence of epilepsy or other conditions requiring an anticonvulsant; presence of thyroid medication for hypothyroidism unless stable on the thyroid medication for 3 months; any current history for an unstable general medical condition or clinically significant abnormal laboratory results. Participants were excluded if taking the following medications: antipsychotic medications, anticonvulsant medications, mood stabilizers, central nervous system stimulants, daily use of benzodiazepines or hypnotics, antidepressant medications, or any other psychotropic medication (including herbal treatments, i.e., St. John's Wort, Omega 3 fatty acids, S-Adenosyl methionine). Finally, participants were excluded if they were receiving or had received vagal nerve stimulation, electroconvulsive therapy, or repetitive transcranial magnetic stimulation, other somatic antidepressant treatments, or psychotherapy in the last 6 months. Usual fMRI exclusion criteria applied including pregnancy or breastfeeding, non-fluency in English, neurological conditions, claustrophobia, or the presence of metal in the body precluding MRI.

Despite these exclusions, there nevertheless remained variation in the quality of the scans: some participants were included in the sample of 37 showing truncated field of view, radiofrequency and field inhomogeneity artifacts. We attempted to examine the extent to which the variation in quality influenced test retest reliability.

### Data Acquisition

Neuroimaging data were collected at four different sites using 3 Tesla scanners: Columbia University (CU); Massachusetts General Hospital (MG); University of Michigan (UM); University of Texas Southwestern (TX). Mean blood-oxygenation-level-dependent (BOLD) T2*-weighted images were then acquired with a gradient echo echo-planar imaging (EPI) sequence during 8-minutes, comprising 240 volumes covering 39 axial slices. Five warm up scans were discarded prior to the recording of BOLD images. T1-weighted Structural 3D axial images were acquired in the same session. Details of the acquisition parameters are displayed in S1 Table.

### Paradigm

**Fig 1:**
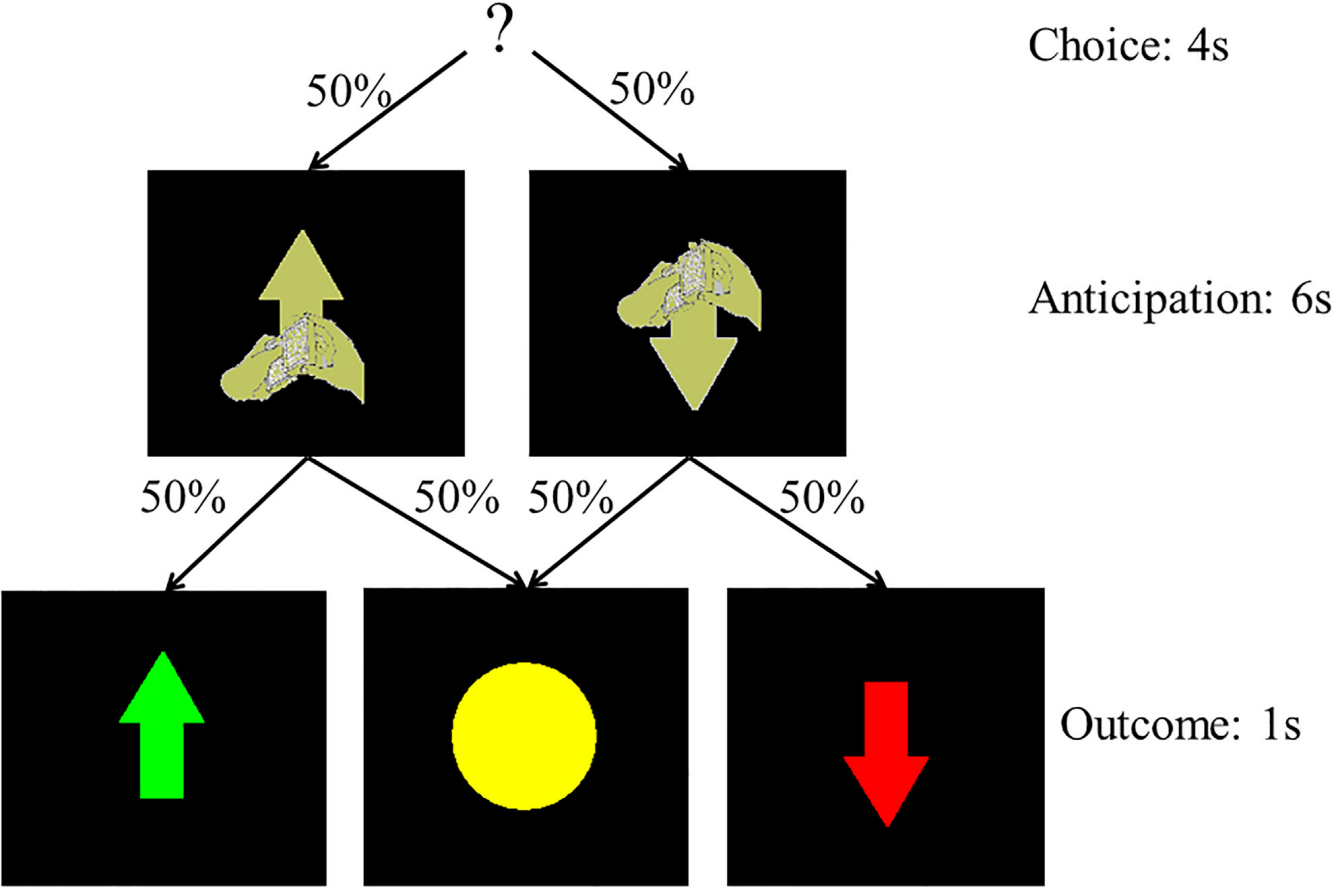
Figure shows the structure of the guessing task, including phases for response, anticipation and outcome (only reward-related feedback shown).

An eight-minute slow event-related card-guessing game was employed (see Fig 1). The task included four possible trial types: the expectation of a possible win, followed by win outcomes (win trials) or no change outcomes (disappointment trials) in equal probability (50%); expectation of a possible loss, followed by loss outcomes (loss trials) or no change outcomes (relief trials) in equal probability (50%). The task constituted one run, in which 24 trials were presented, with 6 trials each for win, disappointment, relief and loss outcomes. Trials were presented in pseudorandom order with predetermined outcomes, and the same task sequence was used in both sessions. Individuals were told that their performance would determine a monetary reward after the scan, with $1 for each win and 50 cents deducted for each loss. Total possible earnings were $3. During each trial, individuals guessed via button press whether the hidden number on the back of a visually presented card would be greater or less than five (4 seconds: presentation of a question mark). An upward or downward arrow was then presented for 6 seconds, representing possible-win or possible-loss respectively, while the participant anticipated the outcome. The outcome then appeared for 1 second (the actual card number for 500ms and then the outcome value for 500ms) followed by a 9 second inter-trial interval (ITI). For outcome value, an up arrow was presented for a $1 win, and down arrow for a 50 cents loss, and a yellow circle was presented for no change outcomes. Individuals practiced the task before the scan.

### Data analysis: fMRI preprocessing and general linear model

Data were preprocessed and analyzed with Statistical Parametric Mapping software, Version-8 (SPM8). Data for each participant were realigned to the first volume in the time series to correct for head motion. Realigned BOLD images were then coregistered with the subject’s anatomical image, after both had been skull stripped using the FSL Brain Extraction Tool (BET). The anatomical image was normalized to the Montreal Neurological Institute (MNI)/ICBM 152 template using a non-linear transformation and segmented into separate tissue types. BOLD images were then transformed to the same space via the segmented structural image (the ‘unified segmentation’ method). Scans with abnormally high or low signal intensity were reconstructed via interpolation using the AFNI 3dDespike tool. The images were then spatially smoothed with an 8mm Gaussian kernel. A first-level fixed-effect model was constructed for each participant. These included four regressors representing different phases of the task: response (4 second duration, starting at the onset of the question mark), anticipation *per se* (6 second duration, starting at the onset of the arrow), outcome (1 second duration, starting at the onset of the number and including the feedback arrow), and baseline (the final 3 seconds of the ITI). The anticipation and outcome regressors were also accompanied by parametric modulators representing reward expectancy (RE) and prediction error (PE) respectively. RE regressors, coupled to the anticipation period, reflected the expected value of the arrow, being set to +0.5 for the up arrow condition (given the 50% chance of winning $1) and -0.25 for the down arrow condition (given the 50% chance of losing 50 cents). PE regressors, coupled to the outcome, were determined by the difference between the outcome and the expected value i.e. +0.5 for a win (1.0–0.5 = 0.5) following an up arrow, -0.5 for no win following an up arrow (0–0.5 = -0.5), +0.25 for a no loss following a down arrow (0-(-.25) = 0.25), -0.25 for a loss following a down arrow (-0.5-(-0.25) = -0.25). Another regressor was included to model omission errors, if these were made, which lasted 17 seconds from the onset of the question mark and replaced other trial events during this period. The Canonical HRF was convolved with each regressor. Movement parameters from the realignment stage were entered as covariates of no interest to control for participant movement. A high pass filter (60 seconds), and autoregressive (AR(1)) modeling were also implemented at the first level.

A first level model constructed in the manner described above was fitted to each voxel using restricted maximum likelihood estimation, for each participant. Each of the two sessions was fitted independently. The resulting parameter maps were analyzed at the second (group) level using directional, voxelwise t-tests. In addition, mean parameter estimates were extracted from regions of interest (ROIs) and focused analyses were performed (see below).

### Data analysis: tests of predictions and reliability

A functionally-defined mask of the ventral striatum (VS) was obtained from a previous study in a separate sample with the same task (identifying activity coupled to PEs) and used for an ROI analysis [22]: the mask was obtained by applying a threshold of p<0.001 uncorrected to the data from all participants in our previous study. The mask was split into separate right and left VS masks; the right VS mask was used as a primary focus for hypothesis testing as effects of interest were clearer in this region in our previous study (see also [23]), but left VS parameter estimates were also examined. Mean parameter estimates from the RE and PE contrasts were extracted from these ROIs.

As a positive control, we used another contrast unrelated to reward processing (anticipation *per se*), and extracted from 8mm spheres located in bilateral visual cortex defined by coordinates from our previous study (-27, -91, 4; 30, -88, 4). In secondary analyses, groups were compared at the whole brain level for all three of the critical contrasts, using a cluster forming threshold of p<0.001 uncorrected and a family-wise error (FWE) rate cluster threshold of p<0.05. Site and slice SNR were included as covariates of no interest in whole brain analyses.

Data from the VS and occipital ROIs were analyzed using t-tests and repeated measures analysis of variance (ANOVA), to examine relevant effects of time, hemisphere, and in the case of VS, contrast type (RE, PE) on the activation within these regions using SPSS (IBM SPSS Statistics Version 20.0). Pearson’s correlations and linear regression were also conducted to assess the relationship between RE and PE parameter estimates in the VS.

The reliability of the data was assessed using the intraclass correlation coefficient (ICC(3,1) [32]). This measure provides a metric of the consistency of data from time 1 to time 2, and is used widely in neuroimaging research [3]. To assess the effect of site, we repeated analyses with and without site as a covariate (for ANOVA) and as independent variables in the regression analyses. For the latter, Columbia University (CU) was taken as the reference site, and three dummy variables were created representing each of the other three sites (the same approach was taken for whole brain SPM analyses). In addition, we tested the consistency of the crucial time by event type (RE, PE) interaction by excluding each site in turn, to determine whether any one site was primarily responsible for the observed effects.

Finally, due to our prediction that VS activation would be unstable across time, it was important to demonstrate that the activity we observe at time 1 is not simply artifactual. To do so, we examine the consistency between the magnitudes of parameter estimates generated in the present study at time 1 and those observed in our previous work with an independent sample [22]. The rationale for this is simple: noise is unlikely to replicate. We computed Bayes Factors [33, 34] between corresponding data reported in the present study and our previous work, to assess the strength of evidence in favor of the ‘null’ hypotheses that the two data sets are generated from a similar set of underlying parameters. Bayes factors of 6 or greater reflect ‘strong’ evidence in favour of a given hypothesis [34], as opposed to ‘anecdotal’ or ‘positive’. In addition, we also assessed the degree of model fit for the relationship between right VS RE and PE in the present data, using the intercept and beta parameters derived from our previous study based in Pittsburgh (henceforth: ‘Pitt’).

### Data analysis: assessing data quality

To assess the effects of data quality on brain activation estimates and their reproducibility, we attempted to generate a single numerical dimension on which to assess variation in data quality. For each participant, measures reflecting SNR and motion were computed (see Table 1). Two measures of whole-brain SNR were computed: slice SNR and voxel SNR [35]. Four measures of motion were computed: maximum motion, mean motion, number of micro-motions (per TR motion >0.1mm), number of macro-motions (per TR motion >0.5mm). As macro motions were not normally distributed across individuals, even after transformation, these were not included in further analysis. These five measures (two SNR; three motion) were computed across both time points, yielding ten variables which were submitted to an unrotated factor analysis. The majority (57.3%) of the variance in these ten measures was explained by a single factor, which was positively associated with motion and negatively associated with SNR, and did not differentiate between the two sessions. The factor explaining the next most variance (12.8%) did not have as obvious an interpretation, although loadings on the different variables seemed to differ slightly between the two sessions. As there were no further factors of theoretical interest, we focused solely on the first factor, and used this as a way to divide the HC cohort into ‘high SNR’ (n = 18) and ‘low SNR’ subgroups (n = 19): all participants with negative loadings were placed in the high SNR group and all with positive loadings were placed in the low SNR group (see Table 1).

**Table 1:** Table describing motion and SNR data, stratified by the two ‘high SNR’ (n = 18) and ‘low SNR’ (n = 19) subgroups.

	Time 1 High SNR	Time 1 Low SNR	Time 2 High SNR	Time 2 Low SNR
Macro Motions: Mean (SD)	0 (0)	1.58 (3.17)	0.17 (0.51)	1.21 (2.57)
Macro Motions: Min-max	0–0	0–11	0–2	0–10
Micro Motions: Mean (SD)	12.89 (13.54)	69.68 (47.35)	21.50 (20.25)	61.21 (36.74)
Micro Motions: Min-max	0–49	6–164	0–75	6–146
Mean Motion (mm): Mean (SD)	0.25 (0.16)	0.86 (0.66)	0.30 (0.17)	0.79(0.91)
Mean Motion (mm): Mean (SD)	0.07–0.72	0.27–3.22	0.11–0.78	0.08–4.34
Max Motion (mm): Mean (SD)	0.48 (0.28)	1.76 (1.07)	0.64 (0.35)	1.54 (1.41)
Max Motion (mm): Min-max	0.15–1.25	0.68–4.81	0.27–1.65	0.19–6.40
Slice SNR: Mean (SD)	306.32 (88.65)	193.57 (68.63)	270.68 (81.04)	220.58 (66.64)
Slice SNR: Min-max	197.93–462.60	93.40–321.59	137.64–407.75	85.07–319.58
Volume SNR: Mean (SD)	69.23 (12.59)	44.95 (9.88)	65.11 (11.69)	46.98 (9.12)
Volume SNR: Min-max	48.85–91.92	25.70–59.66	45.20–84.78	25.67–60.48

Aside from macro-motion variables which were not appropriately distributed, comparisons of transformed motion or raw SNR variables between low and high groups using t-tests were significant in all cases (t(35)>2.06, p<0.047).

Splitting the groups allowed analysis of the data reliability to be conducted separately in the two subgroups. The numerical value of the first factor loading was then inverted, such that data with a higher SNR/less motion had a larger numerical value, and the mean was shifted such that all values were greater than zero. This was done to facilitate interpretation of subsequent analyses of the residuals and model fitting.

We used this variable in several ways. First, we compared variables of interest (e.g. right VS PE responses) between the subgroups, and computed ICCs for each subgroup. Second, as the loading variable was thought to relate to data quality, we examined the relationship between the loading variable and indices of regression model fits including residuals of the fits. The residuals were derived either directly from the regression models or from leave-one-out (LOO) fits of the data. We expected a heteroskedastic relationship between residuals and precision: precisely estimated data should show small residuals, whereas imprecisely estimated data should show variable residuals. Consequently, we used Spearman’s Rho to examine this relationship.

Finally, in a related analysis, we also estimated the best fitting regression model using a weighted least squares procedure: as the loading variable was derived as a proxy of precision, including the variable in a weighted least squares (WLS) model should enhance model fit. However, as the scaling of this relationship was not known (neither its magnitude nor sign), we decided to estimate the scaling parameter by selecting the parameter (using increments of 0.1) which produced the best (log likelihood) fit of the data within the weighted regression model. The scaling parameter was used in the weighted model as an *exponent*, and allowing the best fitting scaling factor to be positive or negative could allow the effect of the parameter to either increase or invert the weighting of the data points. Specifically, the loading factor scores for each participant were raised to the power of the best fitting scaling parameter. Given the combination of the rotation of the loading factor scores and the position of the weighting parameter within the WLS model, we anticipated that a positive scaling factor would fit the data best: larger factor loadings would be associated with more precisely estimated data points. Equally however, if, following fitting, improvement in model fit with the weighted model yielded a negative scaling parameter, we would conclude that scaling of the precision of the data points is exploiting a random or unexplained feature of the data. A negligible parameter would indicate that all data points should be weighted equally. We had no strong expectancies regarding the magnitude of the scaling factor (i.e. whether the loading factor should have a linear or non-linear relationship with weighting).

## Results

### Behavioral performance

Median reaction time was 682.27ms (standard deviation 239.25) at time 1 and 637.27ms (SD 154.10) at time 2. Response omission error rate was low at each time point (time 1: mean 0.35, SD 0.72; time 2: mean 0.35, SD 0.59). No significant differences between the sessions were observed.

### ROI analysis: Ventral striatum

**Fig 2:**
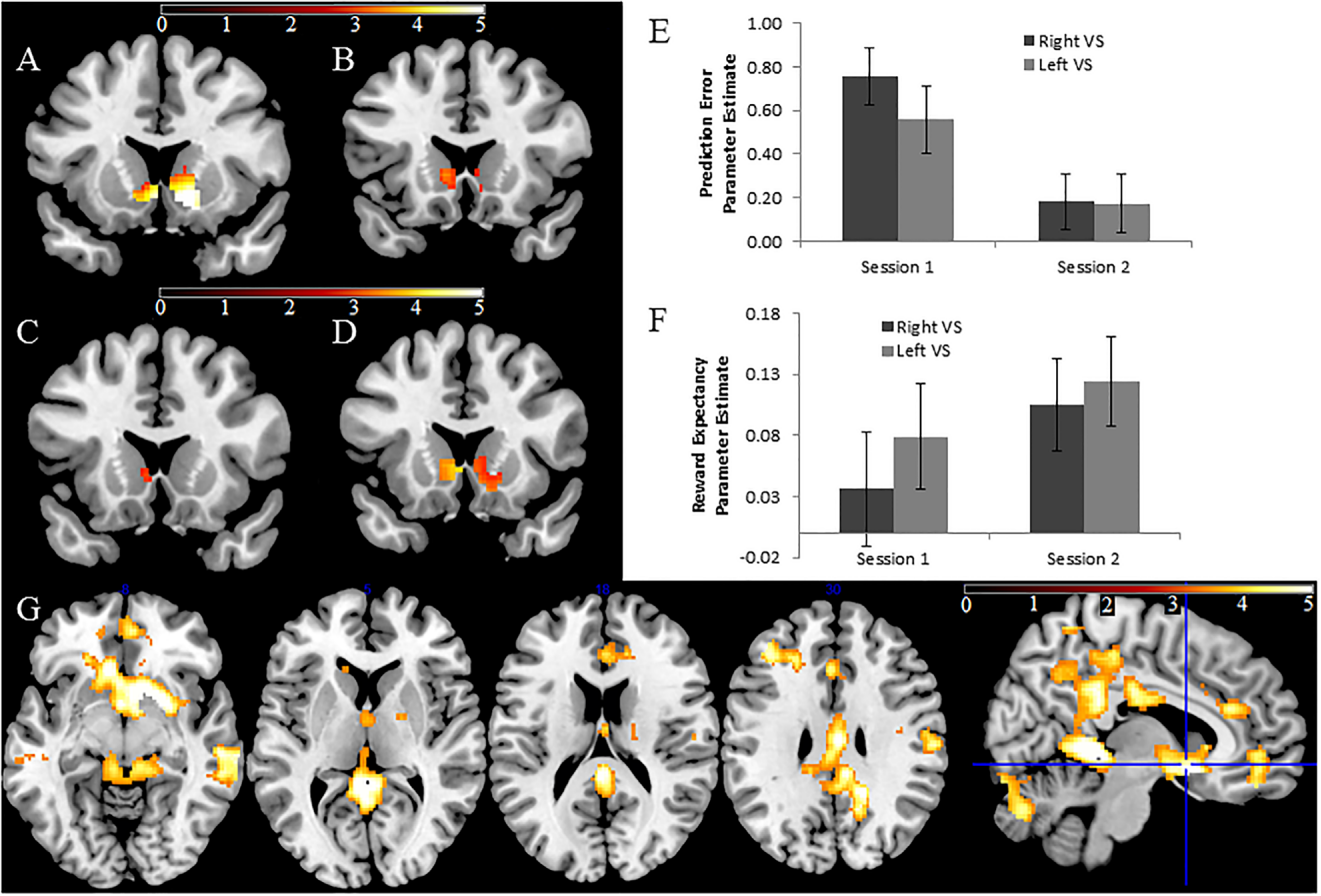
Significant VS PE-related activity was observed at time 1 (A) but not time 2 (B). Significant VS RE-related activation was observed at time 2 (C) but not time 1 (D). Figures thresholded at p<0.025 uncorrected, and masked with the ROI, for display purposes. Bar charts of extracted parameter estimates, displaying the mean PE-related (E) and RE-related (F) findings within the entire VS regions of interest. Error bars reflect standard errors of the mean (SEM). (G). Whole brain PE activations at time 1, thresholded at p<0.001, k>200.

Significant right VS PE-related activation was observed at time 1 (t = 5.65, p<0.001) but not time 2 (t = 1.42, p = 0.17), and a paired t-test revealed that the magnitude of reduction was significant (t = 3.12, p = 0.004). Conversely, significant right VS RE-related activity was observed at time 2 (t = 2.76, p = 0.009) but not time 1 (t<1: see Fig 2), although the difference between the sessions was not significant (t = 1.28, p = 0.21).

**Fig 3:**
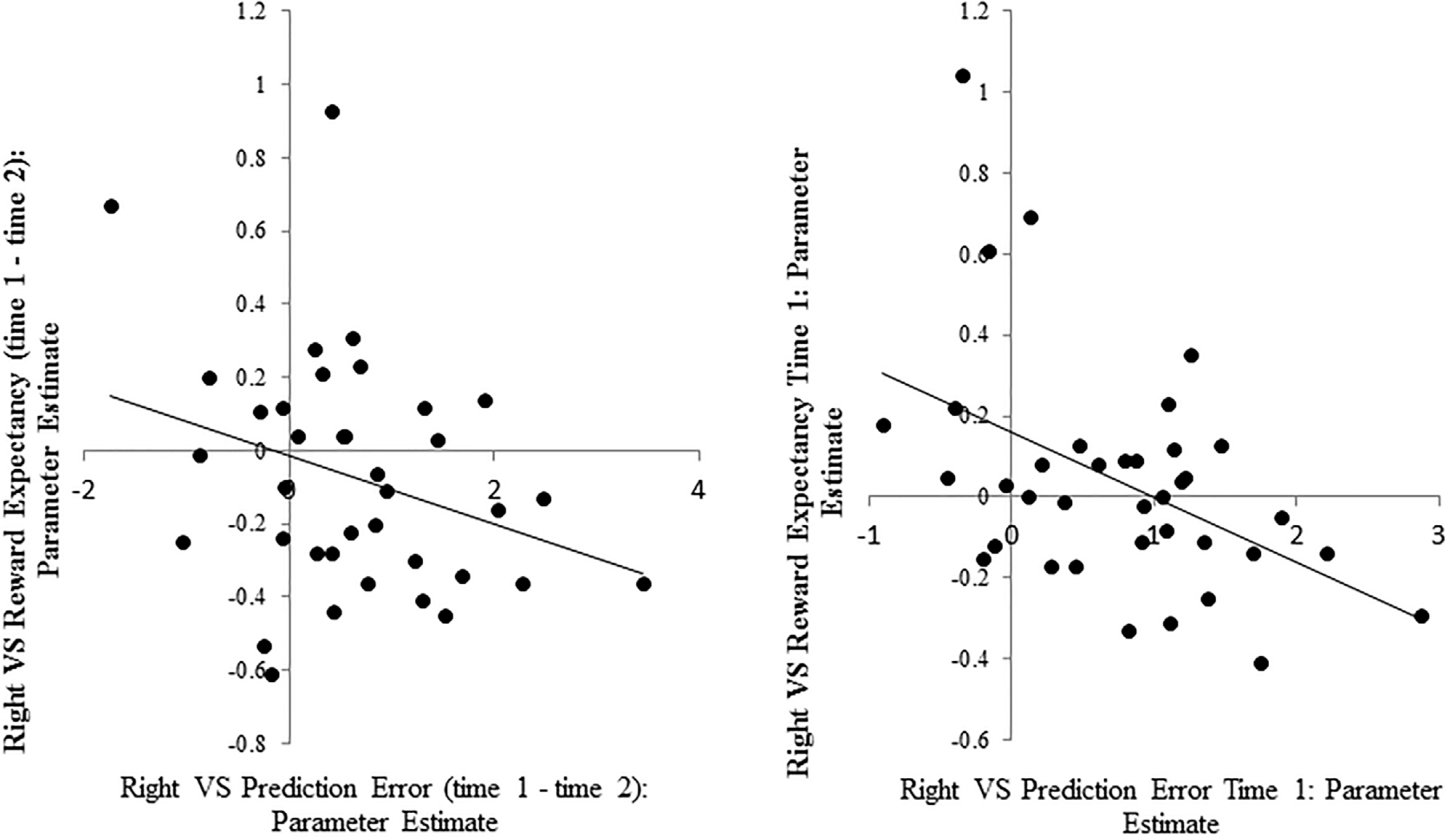
Left: Change in right VS RE activation from time 1 to time 2 is negatively correlated with the change in right VS PE activation from time 1 to time 2. Right: Right VS RE activation at time 1 is negatively correlated with right VS PE activation at time 1.

Across participants, increases in right VS RE-related activity from time 1 to time 2 were associated with decreases in right VS PE-related activity from time 1 to time 2 across participants (r = -0.37, p = 0.025: see Fig 3, Table 2). At time 1 alone, participants with greater right VS RE-related activity had reduced right VS PE-related activity (r = -0.46, p = 0.004; see Table 2). This finding was not observed at time 2 (r = 0.15, p = 0.37). ICCs in right VS were very low: RE—ICC: 0.20; 95% confidence interval -0.13/0.49; F(36) = 1.51, p = 0.11; PE—ICC 0.01 95% CI -0.31/0.33; F(36) = 1.02, p = 0.48.

**Table 2:** Table describing model fit and associated statistics of two regression models applied to key variables of interest (RE/PE) in the present study.

Dependent measure	Right VS RE time 1–time 2	Right VS RE time 1
Independent measure	Right VS PE time 1–time 2	Right VS PE time 1
R^2^ without weighting	0.14	0.21
T (p) without weighting	-2.34 (0.025)	-3.07 (0.004)
T (p) without weighting with site covariate	-2.75 (0.010)	-3.09 (0.004)
Model r^2^ without weighting with site covariate	0.20	0.21
T (p) with weighting	-2.60 (0.013)	-3.07 (0.004)
Best fit weight parameter without site	1.3	0
T (p) with weight with site covariate	-2.94(0.006)	-3.17 (0.003)
Best fit weight parameter with site	1.3	-0.4
Correlation of loading score with squared residuals (rho,p)	-0.35 (0.034)	0.023 (0.90)
Correlation of loading score with LOO squared residuals (rho,p)	-0.36 (0.030)	-0.00 (0.96)
Correlation of loading score with LOO minus normal squared residuals (rho,p)	-0.33 (0.046)	-0.00 (0.99)

Parametric ordinary or weighted least squares regression were used throughout, aside from the association of loading score with the squared residuals generated by the basic model, in which we used Spearman’s Rho.

Data from the left VS followed a similar pattern and were highly correlated with corresponding right VS parameter estimates (r’s>0.58, p<0.001 in all cases), but specific predictions of the TD model, such as a reduction in PE activation from time 1 and time 2, or between-participant correlations between RE and PE measures, did not reach significance. No significant hemisphere effects were observed in an ANOVA reflecting effects of time, hemisphere and condition (F(1,36)<2.77, p>0.11 in all cases).

Given that we had observed poor test-retest reliability of VS activation as assessed by the ICC(3,1) statistic, it became more important to demonstrate that time 1 activations on the task are reproducible, and thus do not represent a spurious finding. To this end, we compared time 1 VS parameter estimates (left/right; RE/PE) obtained in the present study with our previous findings: we observed Bayes factors of between 6.38 and 9.17 in favor of the null hypothesis that there was no difference between the studies. In addition, we compared the relationship between right VS RE and PE data: numerical values of betas and intercepts for the two data sets were very similar (Pitt: beta -1.13, intercept 0.72; EMBARC: -1.32, intercept 0.80). Predicting the present data from parameters (beta/intercept) derived from our previous study yielded a very similar estimate of the amount of variance explained compared to predictions made using the parameters obtained by least squares fitting of the present data (using EMBARC parameters r^2^ = 0.21; using Pitt parameters r^2^ = 0.20).

### ROI analysis: Occipital Cortex

Parameter estimates were obtained from left and right visual cortex ROIs coupled to anticipation *per se*. Occipital activation was significant at time 1 (t’s > 4.79, p’s<0.001) but not time 2 (t’s < 1), and significant differences in overall activation in occipital cortex from time 1 to time 2 (t’s>3.48, p<0.002) were observed. Within an ANOVA model including hemisphere and time factors, no hemisphere effects or interactions were observed (F’s<1.06, p’s>0.31). ICCs in bilateral visual cortex to the anticipation *per se* contrast were higher (left: ICC: 0.52; 95% CI 0.24/0.72; F(36) = 3.17, p<0.001; right ICC: 0.36; 95% CI 0.05/0.61; F(36) = 2.13, p = 0.013).

### Whole brain findings: Prediction error

At time 1, a very large, significant cluster (cluster size 7076; FWE p<0.001; see Fig 2G) was observed, encompassing bilateral VS (peak voxel: 16, 8, -6), thalamus, amygdala, ventromedial PFC and extended ventrally towards the retrosplenial cortex, posterior cingulate and precuneus. A second, large cluster was present within a large section of the cerebellum (cluster size 3564; FWE p<0.001). Other clusters were present in the left superior frontal gyrus (peak voxel -24, 44, 38; cluster size 508; FWE p = 0.001), middle temporal gyrus (peak voxel 66, -28, -10; cluster size 478; FWE p = 0.002), dorsal anterior cingulate cortex (peak voxel 4 32 22; cluster size 374; FWE p = 0.007), the right supramarginal gyrus (PFop/PFt/OP3/OP4: peak voxel 54, -18, 24; cluster size 307; FWE p = 0.015) and finally the right superior parietal lobule (SPL: 7PC; peak voxel 28–46 46; cluster size 247; FWE p = 0.035). Other clusters of activation were observed at uncorrected thresholds. By contrast, no significant or uncorrected activation was observed at time 2.

### Reward Expectancy

No significant or uncorrected activation was observed at time 1. At time 2, no significant clusters were observed, but uncorrected (p<0.001; cluster corrected p = 0.18) activation was seen in the VS (peak voxel: 10, 2, -8). Other uncorrected clusters were also observed in anterior cingulate and anterior insula, and the cerebellum.

### Anticipation per se

Two significant visual cortex clusters were observed at time 1: right occipital (peak voxel: 22, -96, 2; cluster size: 611 voxels; FWE p = 0.001); left occipital (peak voxel: -20, -94, 0; cluster size: 850 voxels; FWE p<0.001). These clusters were close to our *a priori* occipital ROIs, which had been derived from the peak activations in our previous study [22]. In addition, a region of left ventrolateral PFC reached cluster corrected significance (peak voxel: -54, 28, 10; cluster size 364 voxels; FWE p = 0.012). Other uncorrected activations were seen in left parietal and premotor cortex. At time 2, very little activation was observed even at uncorrected thresholds.

### Site effects

We assessed the effect of site in a number of ways. Although significant effects of site were observed in some analyses, the relatively low power of this analysis and large number of potential comparisons meant that the likelihood of spurious effects of site was increased. Our primary goal was to assess the degree to which hypothesized effects were impacted by site effects, and to demonstrate that predicted effect sizes were consistent across sites. To start, we focused on the effect of most interest—the reciprocal increase and decrease in right VS RE and PE activation from time 1 to time 2 (time by event type interaction) as captured by a repeated measures ANOVA. Across all individuals, the effect size (partial eta^2^) was 0.21; if site were included as a covariate, the effect size was 0.23.

The best test of whether site impacts the effect of interest would be to include an interaction between site and the effect in question. This would require testing a three-way site-by-time-by-event type interaction, which this study is underpowered to detect. Instead, to determine whether any one site was responsible for the observed effects, we left each site out in turn and re-ran the primary analyses. Effect sizes varied in a small window from 0.13 to 0.31, despite the removal of one-quarter of the data each time. Site did explain some variance in the ANOVA model as a main effect (F(3, 33) = 3.93, p = 0.017; partial eta^2^ = 0.26), but no two-way interactions with site were significant (all ps>0.081). Regression analyses describing the relationship between RE and PE (see Table 2) were overall very similar with or without the modelling of the effect of site, and hierarchical regression revealed that the addition of site variables did not provide a superior regression model fit given the increase in model parameters. Levene’s test revealed no significant differences in the magnitude of the error variances of any of the variables across sites (F’s<1).

Including site variables in the visual cortex regression analyses slightly reduced the significance of the relationship between time 1 and time 2 activation, although not substantially. As with the VS data, including site in a time by hemisphere ANOVA model led to a main effect of site (F(3, 33) = 5.47, p = 0.004) rather than a significant interaction effect (p’s>0.076). Levene’s test revealed a significant effect of site on the error variance of right visual cortical activation at time 1 (F(3,33) = 3.00, p = 0.044); all other measures were not significant (F’s<1).

The squared residuals generated from all four regression models reported in Table 2 did not significantly differ by site, regardless of how they were computed (standard, LOO), nor was the difference between LOO and standard residuals significantly different.

### Data Quality

Motion variables (micro-motions, mean motion and maximum motion) from each session were log transformed, together with slice and volume SNR for each session. Substantial intercorrelations were seen between the variables, but no clear differences in their magnitude were seen across experiment sessions (see Table 1). A factor analysis was used to generate a data quality ‘loading score’, a scalar variable reflecting data quality, and on this basis to divide the sample into two groups (see Methods): a high SNR group was associated with low levels of motion and high SNR, and the reverse was true for the low SNR group (see Table 1). Moreover, aside from one severe inhomogeneity artifact (time 1) and flickering stripe artifact (time 2), all cases of severe artifacts were in the low SNR group, although individuals with poor coverage were equally represented in both groups. Artifacts of moderate magnitude were also slightly more common in the low SNR group.

### ROI analysis: SNR effects

For PE parameter estimates in right VS, ICCs were similar for low and high SNR groups (High: -0.02; low: 0.04). For RE parameter estimates in right VS, ICCs were higher for the high SNR group (0.30) than the low SNR group (0.12). Left VS ICCs were similar to those of the right VS (whole group: RE: 0.18; PE -0.14; RE: high SNR 0.27 low SNR 0.13; PE: high SNR -0.17 low SNR -0.14). No significant group differences were observed between high and low SNR for left or right VS response to RE or PE (all t’s<1.92, p>0.062). In addition, the null hypotheses that bilateral VS PE activation at time 1, or RE activation at time 2 is equal to zero was independently rejected in both SNR subgroups (all t’s>2.35, p’s<0.032), except the right VS RE activation at time 2, which was at trend-level significance in both groups (t’s = 1.82–2.056, p’s = 0.056–0.085).

There were no significant differences between high and low SNR HC groups in right or left occipital cortex response to anticipation *per se* (t(35)<1), but ICCs were greater in the high SNR group (left 0.64; right 0.43) than the low SNR group (left 0.35; right 0.27). Only the visual cortex ICCs in the high SNR group were significantly different from zero (left: F(17) = 4.50, p = 0.002; right: F(17) = 2.49, p = 0.034), whereas the low SNR group ICCs were not (left: F(18) = 2.07, p = 0.066; right: F(18) = 1.75, p = 0.12).

### Weighted analysis

We compared regression models describing the relationship between right VS RE and PE activation with equivalent weighted least squares models. To generate a weighting, we took the loadings on the motion/SNR factor described previously. These loading scores were subtracted from 4, so that all values were greater than zero and more precise data points were associated with larger values. Given the ordering of the weighting score—more precise variables had the largest numbers—we expected a positive scaling score to be produced in the best fitting weighted least squares model.

Estimation of relationships between RE and PE in the VS were not much affected by reweighting the data points (Table 2), but generally a very slight improvement in fit was seen. However, the best fitting weighting parameter was positive, as expected, for the change in RE and PE from time 1 to time 2. It was negligible but negative for the relationship between RE and PE at time 1.

A second analysis also supported the idea that only the residuals of the relationship of RE and PE from time 1 to time 2 was associated with the data quality loading scores. Specifically, we tested the idea that modelling of inter-subject variability would be worse in participants with noisy data, and thus the residuals of a given model would be associated with the data quality loading score. Association of loading scores with residuals was present in time 1 to time 2 changes in right VS RE and PE, whereby lower quality data was associated with greater residuals, but this relationship was not seen between right VS RE and PE at time 1 (see Table 2). In the former case, the relationship between loading score and residuals was significant not only for conventionally-calculated squared residuals, but also LOO-calculated squared residuals, and also the difference between LOO and conventionally calculated squared residuals. Together, both the weighting and correlation with residuals suggests that residuals—errors of the fit of the relationship between two variables—were calibrated with an independent measure of data precision, but only for the right VS RE/PE time 1/time 2 change scores and not the other relationships.

## Discussion

Dynamic changes in neural activation are consistent with a variety of psychological theories and have been successfully observed in previous fMRI studies [17]. Nevertheless, conventional measures of temporal reliability (e.g. ICCs) cannot distinguish between expected, dynamic changes in regional activation and noisy signal. In the present study, we provide evidence for a methodology to identify expected changes: although reward-related VS activations have very low ICCs over time, they follow the pattern consistent with the temporal difference (TD) models of reward learning. These models hold that following conditioning, reward-related activation should move to the earliest predictor of reward. Thus we observed a significant decrease from time 1 to time 2 in VS-prediction error (PE)- related activation coupled to the outcome, whereas VS-reward expectancy (RE)- related activation coupled to a predictive cue was observed to increase over this time period.

In the present work, we demonstrate that the form of learning predicted by the TD model—a transfer of the *same signal* from outcome- to cue-coupled activation—can be manifest across two testing sessions. Moreover, correlational analyses support the ideas suggested by our previous study [22]: that individual differences in an inferred learning rate seem to play a role in determining variation in VS activations in our paradigm, leading to a significant reciprocal relationship between RE- and PE-coupled VS activation across individuals, but as a consequence, low ICCs. However, to explain the entire pattern of findings, another factor may also be required. PE responses on the second session are somewhat lower than would be expected: due to the 50% contingencies, substantial prediction errors should still be elicited at the second session. By this stage, the outcomes may be perceived as uninformative [36–38] and thus phasic, learning related activation may diminish. This type of effect may accentuate the PE to RE transfer observed across sessions.

Previous examinations of the test-retest reliability of reward related fMRI activations have been inconsistent [11, 39, 40]. Two of the previous reports, employing the monetary incentive delay (MID) task [40] or a similar variant [11] have shown good reliability. In these designs, reward related cues drive motivated responding for reward, and VS activation becomes rapidly coupled to an anticipatory cue (other MID designs, in which there is more variability in outcome value, can drive robust outcome-related activation e.g. [41]). In light of this inconsistency, it should be noted that the dynamic fluctuations predicted by the TD model may not necessarily lead to reduced reliability. For example, if conditioning is rapid, reward expectancy activation may reach asymptote during the first session, leading to a more consistent RE-coupled activation. Its (asymptotic) magnitude may be related to individual differences in a trait-like reward sensitivity [40], which by definition will generalize well from session to session. By contrast, a paradigm more like ours [39], with a guessing component and prediction error-modelled responses, showed low reliabilities—at least for the reward rather than motor-related activations. However, unlike our findings, significant VS PE responses were also seen at the second session, perhaps due to the lack of a predictive cue. Whether reliability is seen or not is likely therefore to depend on the task design, and the interaction with individual differences that determine features of the acquisition curve including the asymptotic response, learning rate and degree of retention from the first to the second time point. Increasing the number of trials per session is also likely to enhance reliability, by mitigating the effects of spurious variability and allowing a more precise estimation of a participant’s beta parameter estimate. An increased number of trials also provide an opportunity for reward expectancy to tend towards asymptote. However, the effect of increasing task length on PE responses is less clear, given that they appear to fall so sharply in the second block. An alternative manipulation that may affect reproducibility is repeatedly to change the contingencies during the paradigm: previous work with probabilistic reversal learning task has suggested that this can be associated with reliable cortical, although not necessarily subcortical, activations [42]. This manipulation may sustain a surprise-related component of PE activations, although higher-order representations of task structure may emerge [43] and render such contingency changes more predictable. Other manipulations which might increase attentional engagement with the task may also enhance reliability [44].

Activation in visual cortex coupled to the anticipation *per se* condition was more reliable, yielding ICCs which compared more favorably with the extant literature [3]. Indeed, these ICCs perhaps underestimated the predictability of the signal, if not its reliability, given that there was a substantial decrease in the amplitude of visual cortex across all subjects, and that ICCs were reduced in individuals with low SNR compared to high. The former point is related to the specific properties of the use of ICC as a measure of reliability: that it is sensitive to changes in variability and range across sessions. The latter point implies that data quality can play a part in determining the upper limit of reliability and accords with previous observations that ICCs can be compromised by even one or two individuals within a group with relatively high motion [45].

The finding that ICCs were generally compromised in lower quality data, but that there was no systematic bias in the estimation of between-participant mean parameter estimates between high and low quality data, suggests that it may be beneficial to weight each datum based on its precision within a group analysis (see e.g. [46, 47]). We took a very simple approach, comparing weighted least squares with ordinary least squares, and found modest improvements of time 1 to time 2 regression model fits (see Table 2). One assumption of weighted least squares is that the measure of precision is known precisely, and this was obviously not met in the present data. Instead, we estimated the scaling parameter that led to the best fitting data. The optimal scaling parameter had the anticipated influence on the weighting of data points reflecting the change in right VS RE and PE from time 1 to time 2: namely that data points (participants) associated with lower SNR and greater motion were relatively down-weighted (Table 2).

Another implication of increased variability in parameter estimates associated with data quality is the possibility that residuals from a between-subjects correlational relationship should scale with precision. Again, this type of relationship was only observed for one of the three relationships we investigated (change in right VS RE and PE from time 1 to time 2: Table 2). We characterized the relationship between residuals and precision further, by re-estimating the regression line and hence the residuals using leave one subject out (LOO) cross-validation. In this case, we would expect participants with particularly noisy data to show even larger residuals, as the least squares fit would somewhat mitigate the effect of mis-estimated data points on the regression line, ‘capitalizing on sampling error’ [48]. The LOO-derived residuals also correlated with our measure of data quality for the change in right VS RE and PE from time 1 to 2. Finally, the difference, per subject, between LOO- and conventionally-derived residuals correlated with data quality only for the RE/PE change association, suggesting that the LOO method indeed may reduce the effect of within-sample fitting bias.

Given that we wish to argue that low levels of reliability of VS activation are indeed expected due to session effects, it becomes more important to show that activity in the VS at time 1 is meaningful. One way to do so is to determine whether it replicates: that is, whether similar data can be obtained at time 1 across separate cohorts. We compared the parameter estimates obtained in our previous study [22] with the equivalent data in the present study. For the VS, the reproduction of parameter estimates was strong, as assessed by Bayes factors [34]. This replication of our previous findings argues against interpretations of the pattern of time 1 to time 2 changes VS activation in terms of regression to the mean, insofar as it would be highly improbable to estimate the same pattern of high and low mis-estimation in two separate cohorts. Moreover, the orthogonalization of the RE and PE contrasts in the design matrix employed is good, allowing each to be independently estimated. In addition, the same relationship between right VS RE and PE was observed at time 1 as had been observed in the previous study. To demonstrate the similarity of parameter estimates, we fitted EMBARC data to the regression parameters (beta, intercept) obtained in the previous study, and explained a very similar proportion of the variance in the data as parameters derived from least squares fitting of the EMBARC data (in contrast to what might be expected by within-sample bias in least squares fitting: [49, 50]). The significance of the relationship between right VS RE and PE at time 1 was slightly but noticeably greater in the EMBARC sample than we had previously reported, probably because we used a more precisely defined ROI for the EMBARC data—one derived from the Pittsburgh sample’s activation rather than a spherical 8mm ROI. Thus, more precise (spatio-temporal) modeling may also contribute to an enhanced power to replicate previous observations.

The general pattern of reproducible activations suggests that equivalent data can be obtained across different data acquisition sites. Our previous study was based in Pittsburgh, whereas there are four data acquisition sites in EMBARC (see Methods). In general, the effect sizes of interest were fairly stable regardless of how site was modeled. In addition, we saw no evidence that site affected the residuals of the regression model fits, which might be the case if one site were associated with unusual data.

Although our previous study [22] did not reveal differences in the magnitude of RE- and PE-related VS activations between healthy individuals and patients with unipolar and bipolar depression, this study did provide corroboration for the view that a TD learning signal is disrupted in depressive illness [29]. The negative relationship across participants between RE- and PE-related activation was similarly present in the control but not patient groups, the latter groups showing a totally uncorrelated relationship despite similar overall RE/PE activation. As pertains to the goals of the EMBARC study, we might predict that patients who are responsive to treatment may begin to show more robust RE-related activation in the second session, while those who do not may paradoxically show a more consistent pattern of low RE- and high PE-related activation. Indeed, enhanced post-treatment anticipation-related activation in depressed individuals was observed by Stoy and colleagues [30]. As we have discussed, individual differences in rate of RE-/PE- transfer (learning rate) also play a significant role in determining the pattern of VS activation, so the extent of remission may also moderate the relationship between RE- and PE- related activation across individuals at the post-treatment session—yielding a negative relationship where previously none existed [22]. The observation that antidepressant treatment might restore a healthy pattern of reward-related VS-activation would provide useful mechanistic support for translational models of depression and related therapeutic strategies [51].

## Summary

We show that inter-individual reward-related activation in the right ventral striatum can be well accounted for if understood and modeled within the framework of temporal difference learning. Thus, low reliability measured by ICC does not necessarily reflect the deleterious effects of physiological noise or poor data quality, although we show that this does have some effect. Anticipation-related visual cortex activation showed greater test-retest reliability, particularly in participants with the best quality data. Finally, weighting data points on the basis of their precision, which we were able to do for the right VS, may be relevant with regard to the clinical use of fMRI. Ideally, any evidence obtained from a patient, even from a sub-optimal scan, should have potential to be clinically useful. Moving toward a clinical framework, we suggest that measurement of RE- and PE- related VS activation in depressed individuals can help elucidate neural mechanisms underlying abnormal temporal difference learning during reward processing in these individuals, and provide neural measures that may serve as future targets for therapeutic interventions.

## Supporting Information

S1 Table Details of structural MRI and BOLD fMRI sequences across sites. (DOCX)
