# Supplementary material for: Accounting for Dynamic Fluctuations across Time when Examining fMRI Test-Retest Reliability: Analysis of a Reward Paradigm in the EMBARC Study
Source: PLoS One. 2015 May 11;10(5):e0126326. doi: 10.1371/journal.pone.0126326 (PMC4427400; doi:10.1371/journal.pone.0126326)
Supplement: S1 Table — (DOCX) [file pone.0126326.s001.docx]

|  | *CU* | *MG* | *UM* | *TX* |
| --- | --- | --- | --- | --- |
| *Scanner* | General Electric 3T | Siemens 3T | Phillips 3T | Phillips 3T |
| *Structural* | FSPGR  TR/TI/TE=6.0ms/900ms/2.4ms  Flip Angle 9°;  FOV: 256x256mm;  Slice Thickness: 1mm;  Matrix: 256x256;  178 continuous slices (4 discarded) | MPRAGE  TR/TI/TE=2300ms/900ms/2.54ms  Flip Angle 9°;  FOV: 256x256mm;  Slice Thickness: 1mm;  Matrix: 256x256;  176 continuous slices | Turbo Field Echo (TFE) sequence  TR/TI/TE=8.2ms/1100ms/3.7ms  Flip Angle 12°;  FOV: 256x256mm;  Slice Thickness: 1mm;  Matrix: 256x256;  178 continuous slices | MPRAGE  TR/TI/TE=2100ms/1100ms/3.7ms  Flip Angle 12°;  FOV: 256x256mm;  Slice Thickness: 1mm;  Matrix: 256x256;  178 continuous slices |
| *BOLD* | TR/TE=2000/28msec;  Flip Angle 90°  FOV=205x205mm;  Slice thickness: 3.1mm;  Matrix 64x64; | TR/TE=2000/28msec;  Flip Angle 90°  FOV=205x205mm;  Slice thickness: 3.1mm;  Matrix 64x64; | TR/TE=2000/28msec;  Flip Angle 90°  FOV=205x205mm;  Slice thickness: 3.1mm;  Matrix 64x64; | TR/TE=2000/28msec;  Flip Angle 90°  FOV=205x205mm;  Slice thickness: 3.1mm;  Matrix 64x64; |

S1 Table: Details of structural MRI and BOLD fMRI sequences across sites.
